# TrackNTrace: A simple and extendable open-source framework for developing single-molecule localization and tracking algorithms

**DOI:** 10.1038/srep37947

**Published:** 2016-11-25

**Authors:** Simon Christoph Stein, Jan Thiart

**Affiliations:** 1 III. Institute of Physics, Georg-August University, 37077 Göttingen, Germany

## Abstract

Super-resolution localization microscopy and single particle tracking are important tools for fluorescence microscopy. Both rely on detecting, and tracking, a large number of fluorescent markers using increasingly sophisticated computer algorithms. However, this rise in complexity makes it difficult to fine-tune parameters and detect inconsistencies, improve existing routines, or develop new approaches founded on established principles. We present an open-source MATLAB framework for single molecule localization, tracking and super-resolution applications. The purpose of this software is to facilitate the development, distribution, and comparison of methods in the community by providing a unique, easily extendable plugin-based system and combining it with a novel visualization system. This graphical interface incorporates possibilities for quick inspection of localization and tracking results, giving direct feedback of the quality achieved with the chosen algorithms and parameter values, as well as possible sources for errors. This is of great importance in practical applications and even more so when developing new techniques. The plugin system greatly simplifies the development of new methods as well as adapting and tailoring routines towards any research problem’s individual requirements. We demonstrate its high speed and accuracy with plugins implementing state-of-the-art algorithms and show two biological applications.

Single-molecule localization microscopy (SMLM) techniques such as PALM^1^ or (d)STORM^2^,^3^ provide super-resolved images beyond the diffraction barrier by exploiting the fact that the center of emission of a single fluorophore’s image on the camera (the point spread function, PSF) can be measured with a much higher accuracy than its width. This is achieved by sequential imaging of a densely populated but sparsely activated set of fluorophores whose locations are determined by automated image analysis. The same computational techniques are employed in single particle tracking (SPT^4^,^5^), where acquired positions are subsequently linked in time to form trajectories of moving probes.

While researchers in these fields today can choose from a plethora of high-performance software suites^6^,^7^, most of them are difficult to extend for specific needs and do not provide a simple way to assess the influence of different parameter inputs. Visualization is usually limited to a display of the final output instead of showing results on a frame-by-frame basis for all intermediate steps. This complicates parameter tuning or the identification of unsuitable settings and obfuscates processing errors. These issues are especially relevant when developing and testing new algorithms.

We present TrackNTrace, the first framework specifically designed for single-molecule localization and tracking applications which implements all processing stages – detecting single particles or patterns, refining their positions and extracting parameters, and subsequent tracking – as user-definable plugins (Supplementary Software). It is designed to allow rapid integration of existing algorithms with minimal effort, including automatic integration into the TrackNTrace graphical user interface (GUI, Supplementary Fig. 1). Each stage’s output can be previewed and inspected in detail using a novel visualization interface, enabling the user to quickly notice odd features and trace the effect of changes in parameters or utilized algorithms, which is not possible with existing programs.

To show the capabilities of the plugin system we include an array of state-of-the-art localization and tracking methods in addition to our own algorithms. As the patterns to detect and track or the observables to extract can be of arbitrary form, TrackNTrace is not limited to standard single-molecule localization applications and can be used in a large number of scenarios such as imaging with engineered PSFs, or cell and microtubule-tip tracking. As an example, we integrated plugins estimating the position and 3D orientation of defocused single-molecule images (ref. 8, Supplementary Fig. 2). To our knowledge this is currently not possible with any other SMLM capable software.

TrackNTrace is implemented in MATLAB, runs on Linux and Windows and can be obtained from its GitHub repository https://github.com/scstein/TrackNTrace.

## Results

### Overview of the TrackNTrace framwork

The framework subdivides the data analysis into four distinct stages:

1. **Correcting raw data:** Measured movies are read and corrected for dark currents and camera artifacts. Analog-digital-converter counts can be converted to photons if a quantitative signal analysis (e.g., maximum-likelihood estimation) is needed.
2. **Detecting candidates:** Potential sources of signal above the background noise are identified in each frame to obtain rough estimates of emitter positions.
3. **Position refinement:** Each candidate’s position estimate is refined to higher accuracy. Commonly, this involves fitting a representation of the microscope’s PSF to a subsection of the frame.
4. **Tracking:** Positions separated in time are connected frame-by-frame to form trajectories. High particle density, intersecting tracks, and re-appearing emitters are the main obstacles to overcome during this stage.

Steps 2–4 are executed by user-definable plugins. Candidate detection currently includes generic image filtering, cross-correlation with a theoretical in-focus PSF or defocused patterns^8^, wavelet filtering^9^, or hypothesis filtering^10^. Refinement can be performed by RadialSymmetry^11^, GPU-GaussMLE^12^, or TrackNTrace’s own routine. The latter is built in C++ using Google Inc.’s *ceres*-*solver* library for fast and accurate model optimization^13^ and supports fitting asymmetric rotated Gaussian PSFs for acquiring 3D positions via astigmatic imaging. TrackNTrace provides a simple and fast nearest-neighbor tracker while more demanding scenarios are handled by u-Track^14^.

**Figure 1:**
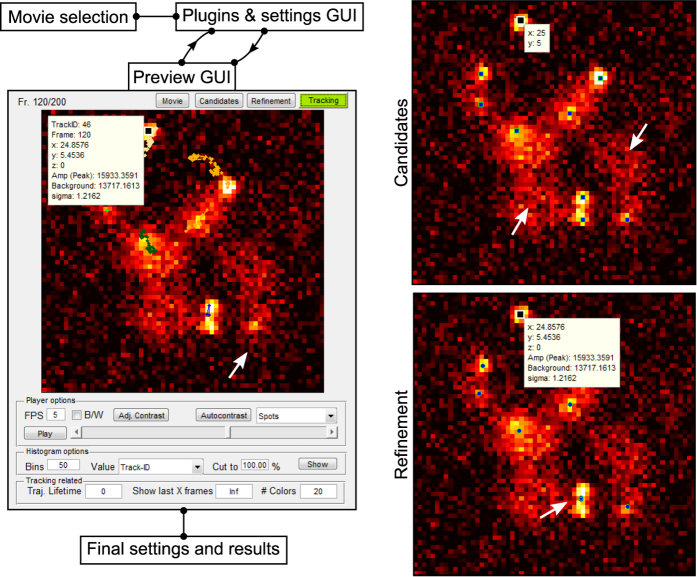
Program flow and visualization interface of TrackNTrace. First, a list of movies or a previously saved settings file is loaded before the main GUI is initialized. There, plugins for steps 2–4 are chosen and their settings adjusted for each movie. At any time during parameter tuning, a preview for an arbitrary part of the current movie can be computed and visualized. The visualizer is able to display the output from all stages on-screen and as a histogram. Selecting a candidate, localization, or track showcases the respective plugin-specific output (e.g., fitted parameter values). Typical issues such as undetected candidates, badly refined positions, or prematurely ending tracks, indicated by the white arrows, can be identified and corrected by choosing different settings. After (repeated) parameter adjustment for all movies, the actual processing starts, saving each movie’s output data along with the chosen settings in a single file.

A preview of (parts of) the input movie can be generated at any time which shows images with overlying identified candidates, refined positions, or tracks as well as all plugin-specific output (Fig. 1). This way, the user can easily spot where a chosen algorithm fails to process emitters correctly and how this behavior changes when parameters are replaced. Final inspection of results is conducted in the same way. We place great importance on this intermediate visualization step for software development and interpretation of scientific data.

After the analysis of a movie is completed the output is saved to a single. mat file which contains the computed results along with information about utilized plugins and their parameters. This file can be loaded by the visualizer or post-processed using various included routines such as drift correction or STORM image generation. The outcome of each processing stage is stored as a 2D-matrix with one column for each output parameter which is easy to export to various file formats for further analysis with external applications.

### Plugin system

TrackNTrace plugins are specified in a single MATLAB script file containing the plugin header and a list of input variables. The header includes, but is not limited to, a plugin’s name, its type (detection, refinement, tracking), parameter and plugin description, and a main function to execute on each frame or the whole position lists. This main function implements the actual algorithm, parses inputs, and returns the output back to TrackNTrace. The list of input parameters consists of type (such as ‘float’, or ‘list’), default value, range, and a tooltip explanation for each parameter. Both description and tooltips are mandatory to clarify what an algorithm does and how it is affected by adjusting these parameters. From this blueprint, a graphical representation of the plugin is built without user intervention. To improve performance, plugin execution is automatically parallelized on a frame-by-frame basis if no temporal information is required and each frame can be handled independently. The plugin creation is designed to be as simple as possible and does not require deeper knowledge of MATLAB or programming in general (see Supplementary Note for an example). A detailed description of the plugin creation is given in the manual shipped with the software.

### Performance evaluation with localization microscopy simulations

**Figure 2:**
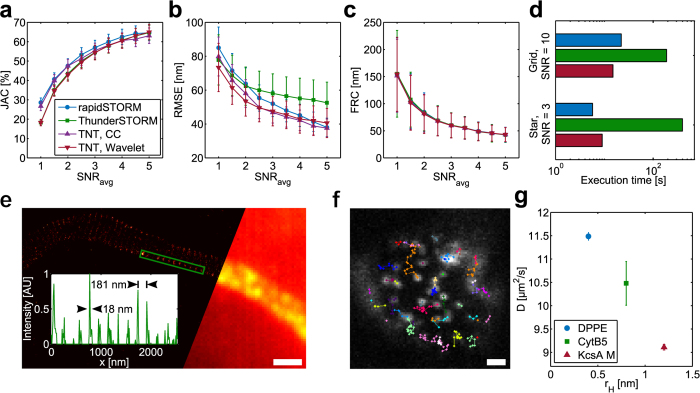
Simulation performance evaluation and experimental results. (**a**–**c**) Overview of simulation results: Jaccard index, root-mean-square error, and Fourier ring correlation of emitters localized with different softwares at various average signal-to-noise ratio (SNR) levels. TrackNTrace is evaluated using both wavelet filtering and cross-correlation for emitter candidate detection. (**d**) Execution time of programs on Siemens star and high-SNR emitter grid data. (**e**) dSTORM imaging of a rat hippocampal neuron axon initial segment shows periodicity of *β*IV-spectrin in the cytoskeleton labeled with Alexa647. Inset: Normalized 1D intensity projection along the rectangle’s wide axis. (**f**) Example image of Atto655-labeled DPPE diffusing in a BLM. (**g**) Diffusion coefficients obtained for lipid bilayer experiments. Scale bar, 1 μm (**e**,**f**).

We evaluated the performance of TrackNTrace with different plugins to demonstrate its usefulness in practical applications using simulated movies (Supplementary Fig. 3), and compared it to established SMLM software. These consisted of rapi*d*STORM, an efficient and popular standalone program^15^, and ThunderSTORM^16^, an ImageJ plugin which performed ahead of competition in a recent comparative meta-study^6^. To this end, we prepared simulated movies of fluorescent emitters distributed on a Siemens star under typical STORM conditions at different signal-to-noise ratios (SNR). The result quality was determined by calculating the Jaccard index (JAC), the root-mean-square deviation from the ground truth (RMSE), and the Fourier ring correlation (FRC^17^). Furthermore we prepared a movie of a fixed numbers on a grid and measured each program’s execution time on this dataset (Fig. 2a–d, Methods).

The Jaccard index which mainly depends on the success of the candidate detection stage indicates that ThunderSTORM and TrackNTrace, both using wavelet-filtering, underperform at low SNR (Fig. 2a). This does not translate into a decrease in resolution as can be seen when comparing JAC and RMSE of rapi*d*STORM which has the highest detection efficiency but loses to TrackNTrace using either cross-correlation or wavelet filtering in terms of RMSE (Fig. 2b). This outcome suggests that a higher number of low-signal localizations tends to have negligible impact on resolution improvement, as has been noted before ref. 18. The structural resolution, however, is unaffected by these differences (Fig. 2c). TrackNTrace is similar to rapi*d*STORM in execution time and even outperforms it at high SNR with emitters placed on a regular grid, while ThunderSTORM is an order of magnitude slower (Fig. 2d, Supplementary Fig. 3). Thus, TrackNTrace fulfills the demands currently imposed upon SMLM software.

### Experimental examples

Next, we tested TrackNTrace in two typical experimental scenarios, one for SMLM and one for SPT (Methods). Figure 2e shows a dSTORM image of an axon initial segment from a fixed mouse hippocampal neuron labeled with an Alexa647-tagged antibody against the N-terminus of *β*IV-spectrin^19^. Whereas the characteristic periodic structure of the axonal spectrin-actin cytoskeletal structure is clearly visible in the reconstructed histogram, it vanishes completely in the widefield projection image. Non-blinking emitters were removed through tracking, further improving image clarity. Performing a Fourier analysis of the line profiles (Supplementary Fig. 4) reveals a fundamental spatial frequency corresponding to a peak-to-peak-spacing of 180–190 nm, the same as previously reported^20^.

To evaluate the particle tracking module, we incorporated lipids and membrane proteins labeled with Atto655 into an artificial black lipid membrane (BLM) spanned over a polytetrafluoroethylene pore 120 μm in diameter. The labeled probes – either DPPE, Cytochrome B5, or a monomeric subunit of the ion channel protein KcsA – were recorded while diffusing through the POPC/POPE BLM and tracked with the u-Track plugin (Fig. 2f,g).

Using the previewer in combination with the very fast nearest-neighbor tracker makes it possible to quickly fine-tune the most important tracking parameters such as maximum particle linking distance or minimum trajectory length which then serve as inputs for the more sophisticated but slow u-Track. This supervised tracking allows us to achieve a higher level of tracking accuracy in a smaller amount of time. The diffusion coefficients obtained by mean-squared displacement analysis (Supplementary Fig. 5) range from 9.1(1) μm^2^ s^−1^ for the KcsA monomer to 11.51(1) μm^2^ s^−1^ for DPPE in excellent agreement with earlier fluorescence correlation spectroscopy measurements^21^.

## Discussion

In conclusion, TrackNTrace combines a strong visual feedback mechanism with simple, straightforward integration of new algorithms to provide a user-friendly environment for accurate and efficient analysis of fluorescence microscopy data. We expect that it will become a highly useful tool for the development of new methods for specific research problems and the comparison to existing solutions. A manual accompanying the software describes the program itself and the plugin development process. We aim to continuously improve the software and invite participation in its development.

## Methods

### Simulation algorithm

Simulations were performed as previously described by Smith *et al*.^22^. Briefly, a Siemens star with *n* = 10 arms was simulated on a 256 × 256 px^2^ grid with a pixel size of *a* = 100 nm (Supplementary Fig. 3). The particle density was fixed to *ρ* = 7.5 px^−1^ and dSTORM-like conditions were emulated by drawing active state durations from a Poissonian distribution (1/*k*_active_ = 1/*k*_deactivate_ + 1/*k*_on_ and *k*_deactivate_ = *k*_off_ + *k*_bleach_) combined with photo-beaching. The time until bleaching was drawn from a geometric distribution with a bleaching probability *p*_bleach_ = *k*_bleach_/*k*_deactivate_. The rates were chosen as *k*_off_ = 1 frame^−1^, *k*_bleach_ = 0.15 frame^−1^, and *k*_on_ = *k*_off_/(5*ρ*) px^−1^. The procedure yielded a list of positions (*μ*_*x*_, *μ*_*y*_) and active state durations per frame *A* ∈ [0, 1].

We assumed an integrated Gaussian point spread function (PSF),


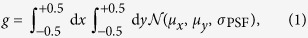


with a size of 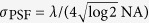. Here, *λ* = 670 nm and NA = 1.4 were chosen. For all active emitters, *A* × *Ng* was added to the image where we assumed a fixed photon yield of *N* = 50. An additional background of 10 photons was added. The end result was distorted by Poissonian noise and normalized to average signal-to-noise ratios of 1 to 5. Each movie had a length of 1500 frames.

To test the execution speed, a 512 × 512 px^2^ frame of 16 × 16 emitters placed at regular intervals next to each other was created in the same fashion. The frame was duplicated 3500 times for a total of 896,000 particles to detect, and normalized to achieve an average SNR of 10. This ensured that all programs would localize the same number of emitters regardless of candidate detection method or initial parameter guesses. The execution speed was corrected for reading the movies into memory. Simulations and performance evaluation, as well as all further data analysis were carried out on a Dell Optiplex 7010 equipped with a Core i7-3770 CPU, 16 GB RAM, and a SSDSC2CT180A4 hard drive running on Windows 7x64.

### Software evaluation

If possible, TrackNTrace, rapi*d*STORM, and ThunderSTORM were evaluated with the same candidate detection and localization criteria, i.e. PSF size, feature size and distance, Maximum-likelihood refinement, fit convergence, or iteration thresholds. The only major differences concerned candidate detection methods (rapi*d*STORM has no wavelet option and was evaluated with a difference-of-averages filter) and PSF model (rapi*d*STORM fits a sampled instead of an integrated Gaussian PSF). The localizations were visually inspected to the best of each program’s capabilities and the best of several runs was chosen.

The output quality was assessed by comparing the obtained set of localizations 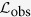 against the known ground truth 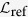 using the Jaccard index (JAC) and the root-mean-square error (RMSE):


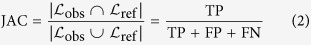



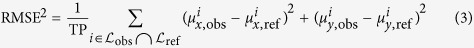


Paired emitters appearing in both sets count as true positives (TP), unpaired ones are either missing from 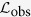 (false negatives, FN) or do not occur in 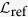 (false positives, FP). The JAC is therefore a measure for how well an algorithm correctly discerns true emitters from background noise whereas the RMSE accounts for the localization precision.

We also calculated the Fourier ring correlation (FRC) for each outcome. The FRC originates from cryo-electron microscopy and has been introduced to the fluorescence microscopy community by Nieuwenhuizen *et al*.^17^. It determines how fast the spatial correlation between two sets of localizations of the same structure declines in Fourier space. Here, the two sets are the ground truth and the observed localizations and the FRC resolution is given as the inverse spatial frequency where the correlation drops to 1/7th of its maximum.

### dSTORM imaging

The experimental details of the dSTORM imaging system and sample preparation have been described in detail before ref. 19. Briefly, the movie analyzed in this work was provided by Melanie Dannemeyer and was recorded from a mouse hippocampal neuron fixed after a maturation period of 11 days *in vitro* (DIV = 11) immunolabeled with a primary OriGene antibody against the N-terminus of *β*IV-spectrin. A secondary antibody (donkey anti-goat IgG) labeled with Alexa647 (Life Technologies) was added and the cells were mounted on a coverslip. An imaging buffer (10 nM TRIS, 100 nM cysteamine hydrochloride, 4.0 mg ml^−1^ glucose oxidase, 0.57 mg ml^−1^ catalase, and 10% glucose) adjusted to a pH of 8.4 was added and the system was excited at *λ* = 647 nm (PhoxX 647-140, Omicron Laserage) in HILO illumination mode using an Olympus IX 71 microscope equipped with a 100× UApoN (Olympus) TIRF-objective with a NA of 1.49, a Di 01-R 405/488/561/635 beam splitter and a FF01 446/523/600/677 emission filter (Semrock). 5000 frames were recorded at a rate of 125 Hz with a DU-885 EMCCD (Andor) at an effective pixel size of 80 nm.

The movie was analyzed using TrackNTrace’s own cross-correlation and nearest-neighbor tracking plugins. Only fitted positions with a signal-to-background ratio above 0.8 and a PSF size of *σ*_PSF_ = 1.42 ± 0.4 px were considered as molecules. Tracks longer than 25 frames were regarded as belonging to fluorophore clusters and discarded. All detected molecule positions were weighted by their localization precision in the final super-resolution histogram which has a pixel size of 10 nm.

### Lipid bilayer tracking

Lipid bilayer diffusion experiments were performed in black lipid membranes (BLM) employing a commercial Bilayer Explorer system (Ionovation). The BLM was created by dissolving a 3 : 2 weight mixture of POPE and POPC in dodecane to a final lipid concentration of 10 mg ml^−1^ and adding it to the Explorer chip containing phosphate buffered saline (137 mM NaCl, 2.7 mM KCl, 1.5 mM KH_2_PO_4_, and 8.1 mM Na_2_HPO_4_) at a pH of 7.4. The bilayer was formed by pumping the solution through a 120 μm polytetrafluoroethylene pore yielding a final bilayer diameter of about 100 μm. We studied DPPE, Cytochrome B5, and the monomeric subunit of the KcsA potassium channel from *Streptomyces lividans*, all of which were expressed, and labeled with Atto655 (AttoTec), as described in detail before ref. 21. They were incorporated via direct addition.

Imaging was performed on a custom-built widefield epi-fluorescence microscope consisting of a *λ* = 637 nm CUBE diode laser (Coherent), a 60×UPLSAPO, NA 1.2 water-immersion objective (Olympus), and an iXon3 DU-860D EMCCD (Andor) with the laser light directed through a BL HC 636/8 cleanup filter, a HC BS 649 dichroic mirror, and a HC 390/482/563/640 emission filter (all Semrock). A *f* = 300 mm lens focused the incoming beam on the back-focal plane of the objective, resulting in a Gaussian excitation spot with 10 μm full width at half maximum. A *f* = 200 mm tube lens in combination with a 3.33× post-magnification system (MAP1030100-A, all lenses by Thorlabs) yielded an effective pixel size of 108 nm. Due to the miniature widefield illumination, the excitation intensity could be set as high as 40 kW cm^−2^, sufficient to photo-bleach molecules in the central field of view fast enough to image non-overlapping emission point spread functions. The acquisition frame rate was 950 Hz.

The movies were analyzed with TrackNTrace’s nearest-neighbor tracker first and the resulting tracks inspected with the visualizer. Particle size, maximum allowed particle-to-particle linking distance, and minimum trajectory length were revised until the outcome was deemed satisfactory. The settings were transferred to the u-Track plugin^14^, which itself optimizes these parameters through forward-backward Kalman filtering, and the final tracks were passed to the mean-squared displacement fit routine (MSD).

Displacement vectors of each trajectory’s *N* position vectors **r**_*i*_ = (*x*_*i*_, *y*_*i*_), *i* = 1, …, *N* were calculated for all possible frame intervals Δ*t*_*ij*_:


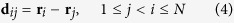



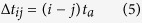


*t*_*a*_ is the camera acquisition time. All displacements {**d**_*ij*_}, *i*−*j* = *k* for one frame interval *kt*_*a*_ were binned into a single, normalized displacement histogram and fit to a Gaussian bell curve:


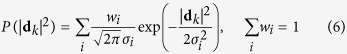


Here, only one diffusion species was allowed so that 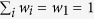. The MSD values *σ*^2^ were subsequently fit to a line to extract the diffusion coefficient *D*:


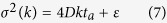


*ε* is a measure for the fit accuracy affected by the localization uncertainty and finite camera exposure time.

## Additional Information

**How to cite this article**: Stein, S. C. and Thiart, J. TrackNTrace: A simple and extendable open-source framework for developing single-molecule localization and tracking algorithms. *Sci. Rep.* **6**, 37947; doi: 10.1038/srep37947 (2016).

**Publisher’s note:** Springer Nature remains neutral with regard to jurisdictional claims in published maps and institutional affiliations.

## Supplementary Material

Supplementary Information
