# Supplementary material for: TrackNTrace: A simple and extendable open-source framework for developing single-molecule localization and tracking algorithms
Source: Sci Rep. 2016 Nov 25;6:37947. doi: 10.1038/srep37947 (PMC5122847; doi:10.1038/srep37947)
Supplement: Supplementary Information [file srep37947-s1.pdf]

Supplementary Information:

TrackNTrace: A simple and extendable open-source framework for developing single-molecule localization and tracking algorithms.

Simon Christoph Stein[1] **and Jan Thiart**[1,*]
[1] III. Institute of Physics, Georg-August University, 37077 Göttingen, Germany
[*] jan.thiart@phys.uni-goettingen.de

List of Figures

1 Example of parameter settings interface. 2
2 Localization and 3D orientation estimation of defocused single molecules. 3
3 Siemens star and grid simulation scenarios 4
4 Fourier spectrum amplitude of line profiles 5
5 Examples of diffusion measurements of lipid bilayer components 6

Contents

1 TrackNTrace plugin system **7**

2 Supplementary software **8**

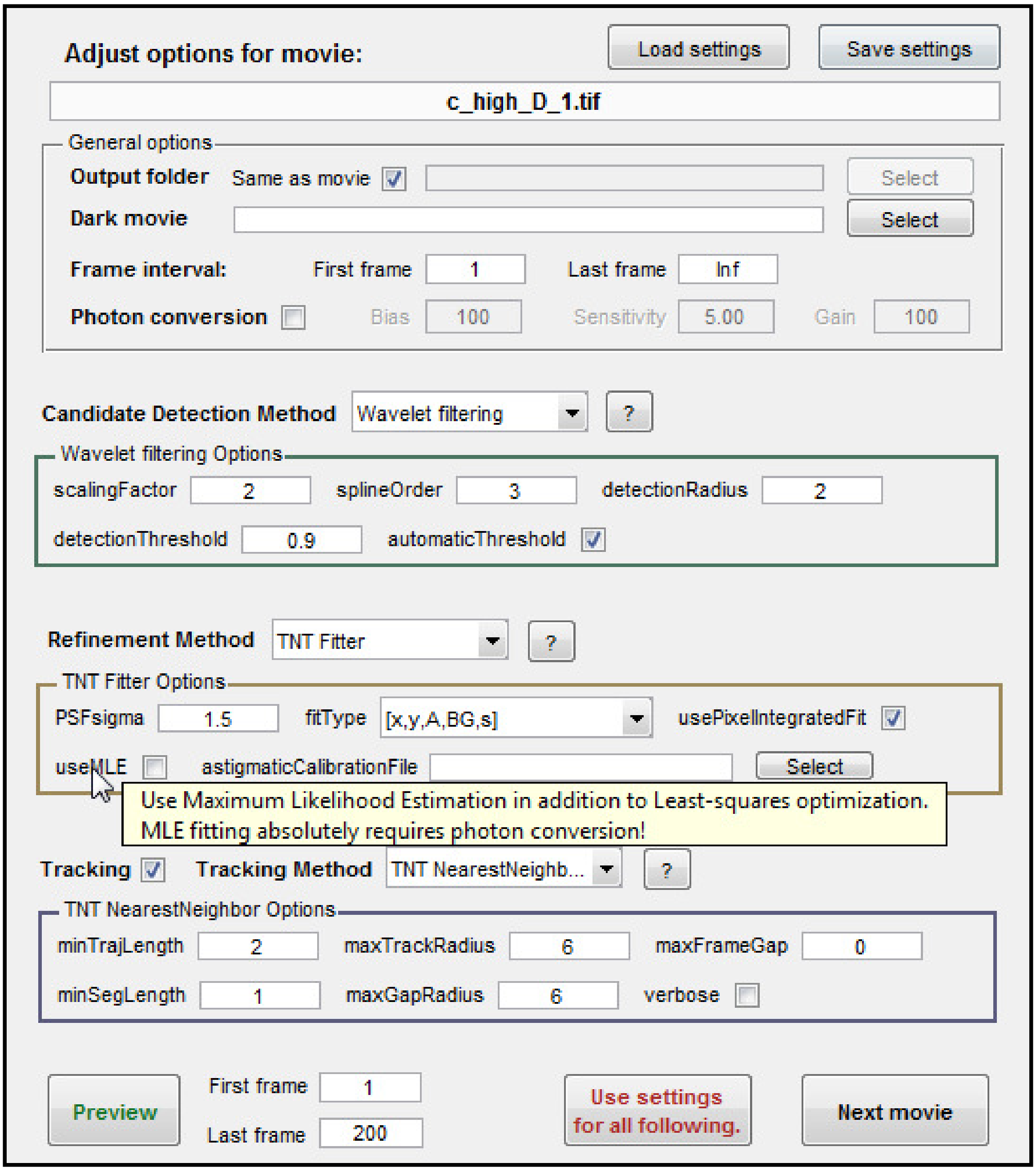

Supplementary Figure 1. Example of parameter settings graphical user interface (GUI). The GUI is rebuilt when choosing a different candidate detection, refinement, or tracking plugin according to the plugin settings. A tooltip is displayed for each parameter when hovering the mouse above. Clicking the Preview button opens the visualizer and lets the user inspect the outcome of the data analysis given the current settings. If satisfied with the current parameter set, the two bottom right buttons will save all settings and either move to the next chosen movie or start processing, or use the same settings for all following movies.

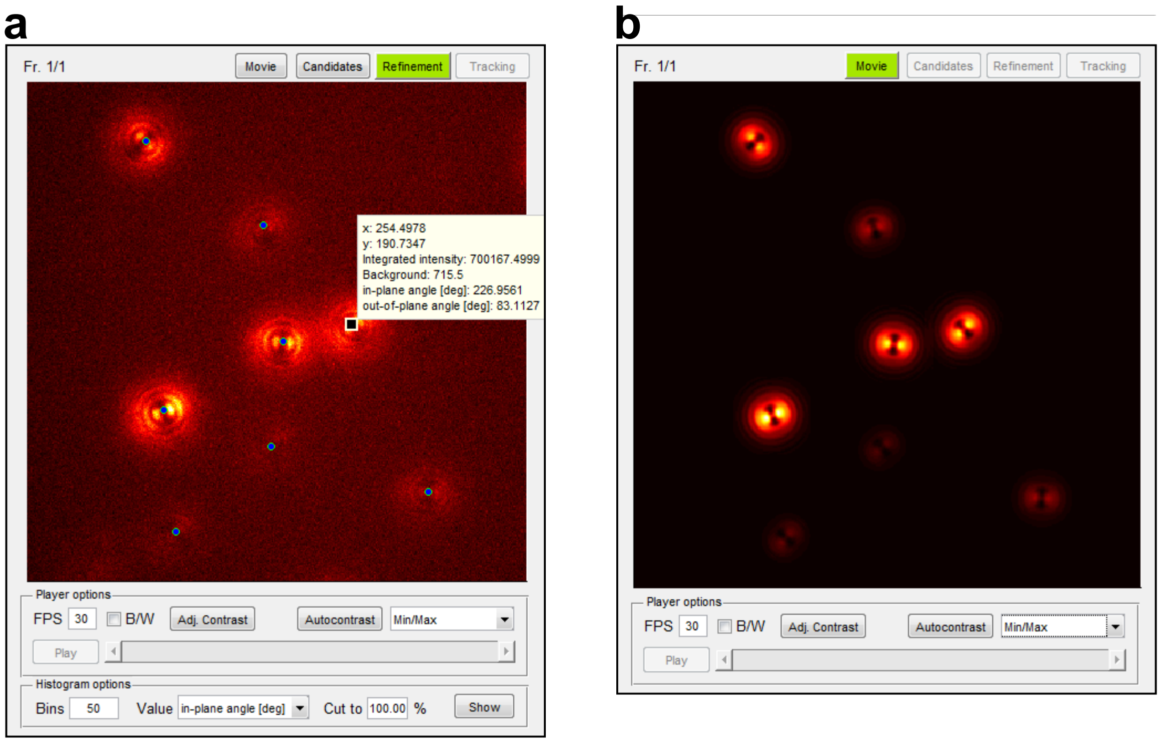

Supplementary Figure 2. Localization and 3D orientation estimation of defocused single molecules. Atto655 molecules were spincoated on a glass surface with a concentration of 0.1 nM and excited with 640 nm at an average power of 0.5 to 1 kW/cm^2. The emission was filtered with a 690/40 bandpass and imaged with a 1.49 NA 100x TIRF objective. The exposure time was 10 seconds with an EM gain of 3 to 5. The defocusing was about 1 micron. Molecules were detected using the `Defocused patterns` candidate plugin and these detections refined to sub-pixel accuracy with the `Defocused refinement` plugin. (**a**) Output of the `Defocused refinement` plugin in the TrackNTrace visualizer. All parameters, like in-plane and out-of-plane angles of the dipole, estimated by the plugin are shown when selecting a detected candidate. (**b**) Computer generated image using the estimated positions, amplitudes and orientations of the detected molecules. This output is generated by the plugin and displayed through the TrackNTrace visualization interface.

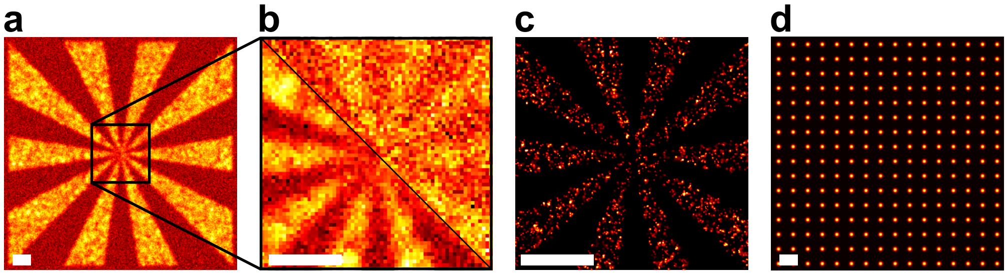

Supplementary Figure 3. Siemens star and grid simulation scenarios. (**a**) Sum intensity image of Siemens star pattern movie at SNR = 3. (**b**) Central region zoom-in displayed for an average SNR of 1 (upper right) and 3 (lower left). (**c**) STORM reconstruction image of same region at SNR 3 in Gaussian rendering style, using TrackNTrace with cross-correlation candidate detection, showing the super-resolved inner spoke pattern. (**d**) Single frame of SNR = 10 simulation grid used for evaluating execution speed. High intensity and regularity allow fixing the number of localizations. All scale bars correspond to 2 µm.

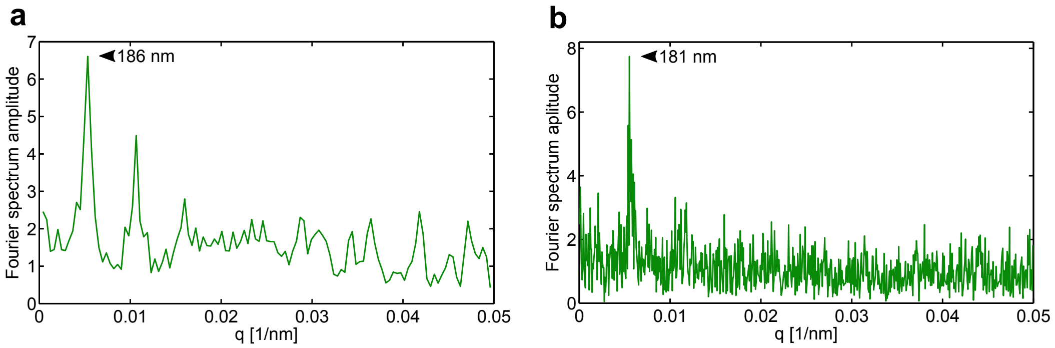

Supplementary Figure 4. Fourier spectrum amplitude of line profiles from **Fig. 2e**. (**a**) Fourier spectrum for line plot of rectangular area from **Fig. 2e**. The first peak with the highest amplitude corresponds to the most significant spatial frequency. Its inverse gives the average spectrin cluster distance, $1/q = 186$ nm. (**b**) Fourier spectrum of line profiles from whole STORM histogram (both edge contours), giving a spectrin cluster distance of $1/q = 181$ nm.

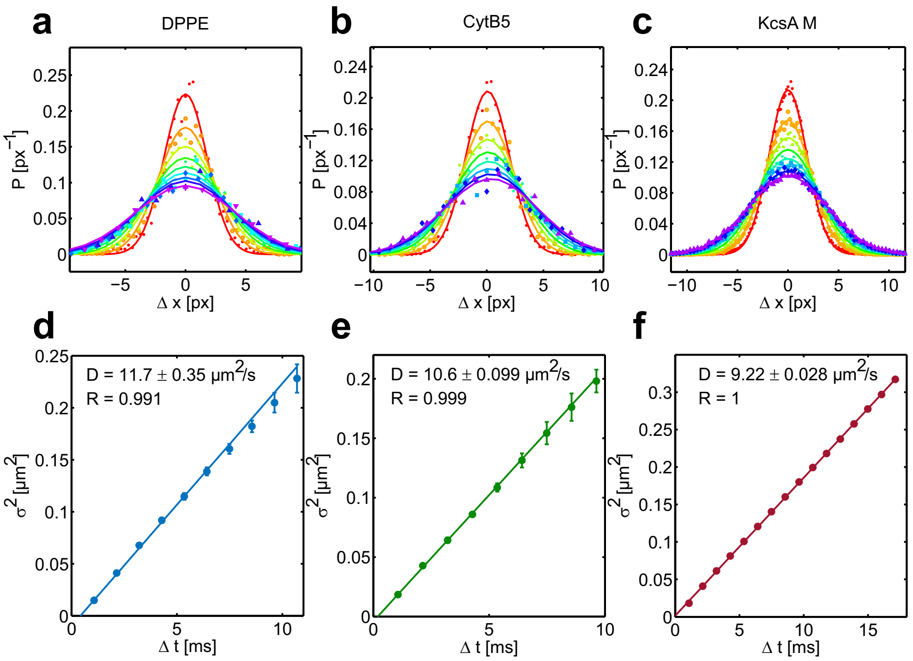

Supplementary Figure 5. Examples of diffusion measurements of lipid bilayer components. (**a**–**c**) Displacement histograms of particle tracks of DPPE, Cytochrome B5, and KcsA Monomer, each obtained from a single movie. Different time intervals are indicated by the color changing from red to violet and start at $\Delta t = 1$ frame. (**d**–**f**) Mean-squared displacement plots obtained from the respective histograms with fits. Inset: Diffusion coefficients and Pearson product-moment correlation coefficients.

Supplementary Note

1 TrackNTrace plugin system

To give an example of the plugin creation process, let us turn the Radial Symmetry (RS) software [1], which is fully written in MATLAB, into a TrackNTrace plugin. The software can be downloaded from the supplementary files of which only the main function, `radialcenter.m`, is needed. The first step is to define the header:

```
function [plugin] = plugin_RadialSymmetry()
name = 'Radial symmetry';
type = 2; % type 2 for position refinement
mainFunc =  @refinePositions_radialSymmetry;
outParamDescription = {'x';'y';'z';'Intens. (Peak)'; 'width'};

% Create the plugin
plugin = TNTplugin(name, type, mainFunc, outParamDescription);

% Description of plugin
plugin.info = ['Particle localization by radial symmetry.\n',...
               'Algorithm by Parthasarathy, NatMet 2012(9).'];

% Add parameters
plugin.add_param('PSFSigma',...
    'float',...
    {1.3,0,inf},...
    'PSF standard deviation in [pixel].');
end
```

The RS algorithm can be used to determine the precise position of an already detected, radially symmetric spot, as such, it belongs to the “refinement” category which has the type number 2. The function `refinePositions_radialSymmetry` implements the actual algorithm on each frame of the movie. Optionally, one can also give a description of the output parameters for plotting purposes as shown here, before invoking the plugin build function. Finally, a mandatory description is added and the input parameters are defined so they can later be set by the user in the TrackNTrace graphical user interface (GUI).

Finally, one needs to add the main function `refinePositions_radialSymmetry` which serves as an interface between the RS function `radialcenter` and TrackNTrace and is called once for every movie frame. The former expects a subimage containing only one particle. The latter provides the movie frame, a list of the position estimates or candidate particles, all input options defined in the above step in the form of a MATLAB `struct`, and the frame index. Thus, the interface function must prepare subimages for each candidate, call `radialcenter`, and save the refined positions:

```
function [refinementData] = refinePositions_radialSymmetry(img,
    candidatePos,options,currentFrameNr)

% Parse input
halfWindowSize = ceil(3*options.PSFSigma);
refinementData = zeros(size(candidatePos,1),5);

for iCand = 1:size(candidatePos,1)
    % Define subimage
    posXY = candidatePos(iCand,1:2);
    idxX = posXY(1)-halfWindowSize:posXY(1)+halfWindowSize;
    idxY = posXY(2)-halfWindowSize:posXY(2)+halfWindowSize;

    % Call Radial Symmetry function
    [xc,yc,sigma] = radialcenter(img(idxY,idxX));

    % Transform relative coordinates back to image coordinates
    xc = xc+posXY(1)-(halfWindowSize+1);
    yc = yc+posXY(2)-(halfWindowSize+1);
    zc = 0;

    % Save data
    refinementData(iCand,:) = [xc,yc,zc,img(round([yc,xc])),sigma];
end

end
```

The size of the subimages is determined by the input parameter `PSFSigma` as defined in the header. The rest of the function is straightforward: After allocating the output memory, the function loops over every candidate, calculates the subimage coordinates, calls `radialcenter`, and converts back to image coordinates before saving the output.

The input and output data structure is rigid, hence the function declaration, handling of input variables, and the number of output variables has to follow a certain standard laid out in the software manual and the accompanying plugins. In most cases, software code like the above example can be assembled within minutes by copying existing code with some minor modifications.

2 Supplementary software

TrackNTrace is implemented in MATLAB and currently runs on Linux and Windows. It can be obtained from its GitHub repository https://github.com/scstein/TrackNTrace.

References

[1] Parthasarathy, R. Rapid, accurate particle tracking by calculation of radial symmetry centers. *Nat. Methods* **9**, 724–726 (2012).
